# The impact of the six pillars of lifestyle medicine on the biology of skin aging

**DOI:** 10.3389/fragi.2026.1822471

**Published:** 2026-06-17

**Authors:** Jaime Piquero-Casals, Adriana R. Cruz, Lucas Ponti-Concetti, Ludmila Prudkin, Anthony Brown, Mónica Foyaca, Eduardo Rozas-Muñoz, Juan Francisco Mir-Bonafé, Daniel Morgado-Carrasco, Thierry Passeron

**Affiliations:** 1 Department of Aesthetic Dermatology and Laser, Dermacentric Health & Aesthetics, Barcelona, Spain; 2 Medstar Shah Medical Group, MD, United States; 3 Department of Dermatology, Amuna Vitality, Madrid, Spain; 4 Innovation and Development, ISDIN, Barcelona, Spain; 5 Department of Dermatology, Hospital San Pablo, Coquimbo, Chile; 6 Department of Dermatology, Hospital Son Llàtzer, Palma de Mallorca, Spain; 7 Department of Dermatology, Hospital Clínic de Barcelona, Universitat de Barcelona, Barcelona, Spain; 8 Department of Dermatology, Centre Hospitalier Universitaire de Nice, Université Côte d'Azur, Nice, France; 9 Centre Méditerranéen de Médecine Moléculaire, INSERM U1065, Université Côte d'Azur, Nice, France

**Keywords:** exercise, exposome, lifestyle medicine, nutrition, pollution, psychological stress, senescence, skin aging

## Abstract

Skin aging arises from the interaction between intrinsic genetic programs and cumulative environmental and behavioral exposures collectively defined as the exposome. Increasing evidence indicates that core molecular pathways driving skin aging, including mitochondrial dysfunction, oxidative stress, chronic inflammation, cellular senescence, and extracellular matrix remodeling are responsive to modifiable lifestyle factors. This narrative review examines the mechanistic and translational evidence linking the six pillars of Lifestyle Medicine; nutrition, physical activity, stress regulation, sleep, avoidance of toxic exposures, and social connection, to fundamental biological processes involved in skin aging. Across experimental models and human studies, these lifestyle domains converge on established hallmarks of aging, modulating redox balance, inflammatory signaling, mitochondrial biogenesis, epigenetic regulation, DNA repair, and dermal matrix integrity. Nutritional patterns influence glycation and oxidative stress; physical activity enhances mitochondrial and vascular function; chronic stress and sleep disruption amplify neuroendocrine and inflammatory pathways; toxic exposures activate matrix-degrading and senescence-associated cascades; and social isolation is associated with heightened systemic inflammatory tone. Collectively, these findings support the concept that skin aging represents a biologically plastic process shaped in part by lifestyle-dependent modulation of conserved aging mechanisms. Integrating lifestyle-responsive pathways into skin aging research may enable more targeted, preventive, and mechanism-based strategies in dermatological practice.

## Graphical Abstract

**Figure:**
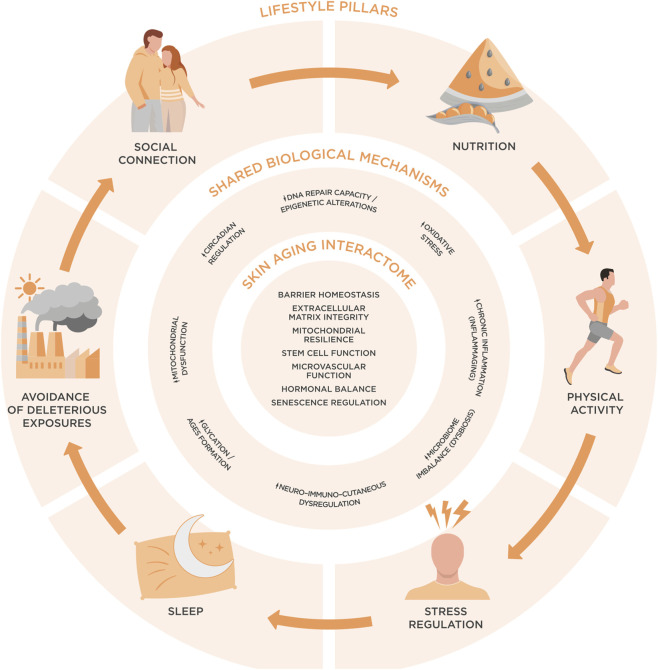
The six pillars of Lifestyle Medicine converge on shared biological pathways within the skin aging interactome, including oxidative stress, chronic inflammation, mitochondrial dysfunction, impaired DNA repair, and extracellular matrix remodeling. Through cumulative exposome and behavioral influences, these mechanisms interact to regulate key processes such as barrier homeostasis, dermal integrity, vascular function, and cellular senescence, ultimately shaping skin aging trajectories.

## 1 Introduction

Skin aging is increasingly viewed as a multifactorial process in which intrinsic programs interact with lifelong environmental and behavioral exposures (the exposome). Photoaging is primarily defined as skin aging driven by chronic ultraviolet (UV) exposure and represents the main extrinsic determinant of wrinkles, pigmentary changes, and dermal matrix degradation. In contrast, intrinsic aging reflects genetically programmed, time-dependent changes, although both processes converge on shared molecular pathways such as oxidative stress, inflammation, and epigenetic regulation. Ultraviolet radiation, air pollution, tobacco smoke, psychosocial stress, nutrition, and daily habits modulate mitochondrial function, redox balance, and epigenetic regulation, progressively shaping the skin aging phenotype (Papaccio et al., 2022). Consistent with this, impaired antioxidant defenses and age-related mitochondrial decline contribute to dermal and epidermal deterioration (Curtis et al., 2015). Beyond cutaneous endpoints, lifestyle-oriented interventions, including psychological and relational approaches, have shown clinically meaningful improvements in quality-of-life outcomes in chronic inflammatory disease, underscoring the translational relevance of modifiable determinants (Tsoi et al., 2024). The skin interactome framework integrates genome, microbiome, and exposome as interacting regulators of skin aging biology (Khmaladze et al., 2020). This framework also encompasses additional domains such as microbiome dynamics, hormonal aging (e.g., menopause-related changes), and circadian regulation beyond sleep duration, which interact with lifestyle-related exposures and modulate key pathways involved in skin aging. Recent models further emphasize the role of the skin and gut microbiome in immune regulation and cutaneous homeostasis (Merra et al., 2024). This aligns with emerging evidence supporting an epithelial barrier-centered paradigm, in which chronic environmental stressors, including pollution, detergents, dietary additives, and microbiome perturbations, can disrupt epithelial and immune pathways relevant to skin integrity and aging (Pat et al., 2024). Epidemiologic estimates suggest that approximately 80% of visible skin aging is driven by extrinsic influences (Musumeci et al., 2022; Farage et al., 2008). In parallel, large-scale analyses indicate that environmental and lifestyle exposures explain a substantially greater fraction of aging-related mortality variation (∼17–19%) than polygenic risk scores (<2%), reinforcing the clinical importance of modifiable exposures in biological aging trajectories (Argentieri et al., 2025).

Lifestyle Medicine provides a structured framework to operationalize these determinants. Core pathways central to skin aging, including mitochondrial respiration, oxidative stress, extracellular matrix remodeling, and senescence-associated secretory activity are responsive to lifestyle-related inputs (Oizumi et al., 2024), and the discipline is supported by an extensive evidence base linking diet, physical activity, sleep, and stress regulation to health outcomes (Rippe, 2023). Increasing evidence further shows that these domains can influence molecular and phenotypic markers of aging (Berisha et al., 2025) and may measurably alter biological aging metrics captured by modern aging clocks (Uribarri et al., 2010). Advances in longevity science highlight mitochondrial efficiency, cellular senescence, and systemic inflammatory tone as key determinants of biological aging across tissues, mechanisms also implicated in skin aging (Min et al., 2024).

While previous reviews have addressed intrinsic and extrinsic determinants of skin aging and exposome-related mechanisms, the present work is distinguished by its application of a Lifestyle Medicine framework, organizing evidence around six clinically actionable domains that converge on core biological pathways of aging. Building on this framework, we examine mechanistic and translational links between these domains and the biology of skin aging, positioning Lifestyle Dermatology as a preventive, mechanistically grounded complement to dermatologic practice.

## 2 Methods

We performed a narrative (non-systematic) review of the literature. PubMed, Scopus, and Web of Science were searched from inception to December 2025. The search strategy combined controlled vocabulary (e.g., MeSH terms) and free-text terms related to skin aging and lifestyle factors. The core search string used in PubMed was: (“skin aging” OR “cutaneous aging” OR photoaging) AND (“lifestyle” OR “lifestyle medicine” OR nutrition OR diet OR exercise OR “physical activity” OR sleep OR “psychological stress” OR smoking OR pollution OR exposome OR “social determinants”) AND (“oxidative stress” OR inflammation OR inflammaging OR senescence OR “extracellular matrix”) Equivalent adaptations of this strategy were applied in Scopus and Web of Science. Inclusion criteria comprised: (i) original research articles, clinical studies, and relevant reviews; (ii) studies addressing mechanistic pathways, clinical manifestations, or preventive strategies related to skin aging; and (iii) publications in English. Exclusion criteria included: (i) studies not directly related to skin biology or aging processes; (ii) articles lacking mechanistic or clinical relevance; and (iii) non-peer-reviewed sources. In cases where skin-specific data were lacking, studies focusing on other epithelial tissues or systemic aging mechanisms were appropriately included to support mechanistic extrapolation. Study selection was based on relevance to the scope of the review, with priority given to mechanistic studies, translational research, and high-quality clinical or epidemiological evidence. Titles and abstracts were screened, followed by full-text evaluation when appropriate. Additional articles were identified through manual screening of reference lists of selected key articles and authoritative reviews. This process involved reviewing cited references for relevance to the predefined thematic domains (mechanistic pathways, clinical manifestations, and preventive strategies), and including those that met the inclusion criteria. Given the narrative nature of the review, no formal quality scoring or PRISMA-based selection process was applied.

## 3 Findings and discussion

Lifestyle Dermatology adopts a systems-oriented model in which the six lifestyle pillars converge on molecular pathways central to skin aging. Building on the skin interactome framework integrating genomic, microbial, and exposome influences, this perspective highlights how modifiable behaviors regulate oxidative stress, inflammatory signaling, mitochondrial function, DNA repair, extracellular matrix remodeling, and barrier integrity. The mechanistic effects of each lifestyle pillar are summarized in Table 1, and their convergence within the skin aging interactome is illustrated in the Graphical abstract image. Conceptually, these mechanisms can be understood as a stepwise process in which exposome-related and lifestyle factors influence systemic pathways, including oxidative stress, inflammation, and metabolic regulation, ultimately translating into skin-specific structural and functional changes.

**TABLE 1:** Lifestyle pillars and their mechanistic effects on skin aging biology.

Lifestyle pillar	Primary mechanisms affected	Key findings/evidence	Cutaneous outcomes
Nutrition	↓ oxidative stress, ↓ glycation, modulation of microbiome	Polyphenols ↓ NF-κB; Mediterranean diet ↓ inflammation	Improved collagen integrity, barrier function
Physical activity	↑ mitochondrial biogenesis, ↑ microcirculation, ↓ inflammation	Aerobic exercise ↑ skin perfusion 8×; IL-15 ↑ mitochondrial function; SIRT3↑	Improved dermal remodeling, hydration
Stress regulation	↓ cortisol, ↓ IL-6/CRP, normalized HPA axis	Stress ↑ IL-6/CRP; chronic stress ↓ collagen synthesis	Reduced inflammaging, better repair
Restorative sleep	↑ Nocturnal repair, ↓ oxidative stress	Poor sleep ↑ TEWL, ↓ hydration; sleep deprivation ↑ inflammation	Improved barrier integrity
Avoidance of deleterious exposures	↓ ROS, ↓ MMP activation, ↓ DNA damage	Smoking ↑ MMP-1; ozone ↑ lipid peroxidation, AhR stimulation Sun exposure ↑ DNA damage, inflammation and MMP-1; ↓ collagen production; promotes cell senescence and dyschromia	Reduced extrinsic aging
Social connection	↓ chronic inflammation, ↓ cortisol	Loneliness ↑ IL-6/CRP and cortisol responses	Improved aging resilience

### 3.1 Pillar I: nutrition and skin aging

Nutrition is a major modifiable determinant of skin aging and represents a key interface between systemic metabolism and cutaneous biology. Dietary patterns influence mitochondrial efficiency, inflammatory signaling, and epigenetic regulation—core mechanisms governing skin aging trajectories (Haykal et al., 2025). Diet also reshapes the gut microbiome, generating metabolites that modulate systemic inflammation and aging biology (Curtis et al., 2015).

Metabolically, dietary exposures broadly diverge into patterns that promote glycemic instability, oxidative stress, and inflammation versus those supporting metabolic homeostasis and barrier integrity. High-glycemic diets and cooking practices rich in advanced glycation end products (AGEs) accelerate the formation of these compounds (Uribarri et al., 2010), contributing to collagen cross-linking, increased stiffness, and impaired extracellular matrix function. Polyphenols reduce lipid peroxidation and NF-κB–mediated cytokine release in dermal cells (Papaccio et al., 2022), while omega-3 fatty acids shift eicosanoid profiles toward less inflammatory mediators, supporting matrix remodeling. Observational data consistently associate Mediterranean-style dietary patterns, rich in fruits, vegetables, legumes, fish, and olive oil, with fewer wrinkles and reduced severity of clinical features commonly associated with photoaging (Gantenbein and Kanaka-Gantenbein, 2021; Purba et al., 2001; Latreille et al., 2012; Hughes et al., 2021). Notably, monounsaturated fats from olive oil, but not animal sources, are associated with reduced severity of UV-related skin aging features (Purba et al., 2001; Latreille et al., 2012; Hughes et al., 2021). These effects are particularly relevant in photoaging, where ultraviolet-induced reactive oxygen species amplify inflammatory signaling and matrix degradation.”

Interventional data further support mechanistic plausibility. Carotenoid-rich foods increase cutaneous carotenoid levels and correlate with reduced oxidative stress *in vivo*. Certain bioactive compounds modulate epigenetic pathways linking environmental exposures to gene expression; for example, broccoli-derived compounds inhibit histone deacetylases (HDACs), while garlic-derived diallyl-disulfide increases histone acetylation (Alegría-Torres et al., 2011), mechanisms associated with regulation of inflammation, keratinocyte function, and extracellular matrix remodeling relevant to skin aging. Lifestyle behaviors also cluster, as improvements in exercise, sleep, and stress regulation are associated with better dietary quality (Fischer and Ouyang, 2025).

Within the gut–skin axis, diet-induced dysbiosis may impair intestinal barrier function, leading to increased gut permeability and translocation of microbial products such as lipopolysaccharide (LPS) into the circulation. This process promotes systemic low-grade inflammation, a key driver of aging-related pathways, which is associated with increased matrix metalloproteinase (MMP) activity and extracellular matrix degradation in the skin. In contrast, fiber- and polyphenol-rich diets enhance short-chain fatty acid production, which supports intestinal barrier integrity and immune regulation (Merra et al., 2024). Short-chain fatty acids (SCFAs), particularly butyrate, acetate, and propionate, exert anti-inflammatory effects by modulating regulatory T-cell activity and suppressing NF-κB–mediated cytokine production. Butyrate also enhances epithelial tight-junction integrity and promotes barrier homeostasis through histone deacetylase inhibition and improved mitochondrial function. Through these mechanisms, SCFAs contribute to the regulation of systemic inflammatory tone and may indirectly support epidermal barrier integrity and extracellular matrix preservation relevant to skin aging. Through these mechanisms, dietary patterns influence systemic inflammatory tone, which in turn can affect skin biology by promoting oxidative stress, extracellular matrix degradation, and impairment of skin barrier function. Conversely, chronic excess of saturated fats and refined sugars promotes gut dysbiosis, intestinal permeability, and systemic inflammation, indirectly contributing to skin aging processes (Musumeci et al., 2022).

Clinical trials suggest that selected probiotics may improve wrinkle depth and elasticity, potentially through modulation of systemic inflammation, reduced circulating lipopolysaccharide (LPS) levels, and downstream effects on MMP activity and barrier integrity (Challa et al., 2025). However, evidence remains limited, and specific microbial taxa or diversity patterns associated with these effects are not yet fully characterized.

Collectively, these data indicate that dietary exposures influence the biochemical and inflammatory milieu in which skin aging progresses (Hauser et al., 2022). While ultraviolet radiation remains the primary driver of photoaging, dietary factors may modulate the biological response to UV-induced damage by influencing oxidative stress, inflammatory signaling, and epigenetic regulation, thereby modulating the skin’s response to environmental damage.

### 3.2 Pillar II: physical activity and dermal remodeling

Regular physical activity modulates key pathways implicated in skin aging, including mitochondrial function, vascular responsiveness, extracellular matrix turnover, and systemic inflammation. In controlled human studies, acute aerobic exercise increases cutaneous blood perfusion by up to eightfold (Oizumi et al., 2024), enhancing nutrient delivery and thermoregulation. Age-related declines in microvascular responsiveness can be partially reversed with consistent moderate training, improving nitric oxide–mediated vasodilation and supporting hydration. Exercise also induces neuromodulators such as endorphins and endocannabinoids, contributing to stress resilience (Núñez-Cortés et al., 2025).

Beyond acute effects, regular physical activity attenuates several hallmarks of aging, including mitochondrial dysfunction, inflammaging, stem cell exhaustion, and extracellular matrix remodeling, and functions as a peripheral circadian regulator supporting tissue homeostasis (Qiu et al., 2023; Su and Xiang, 2025). Endurance exercise promotes IL-15–mediated mitochondrial biogenesis and reduces reactive oxygen species accumulation (Jia et al., 2023). In murine models, treadmill training increases dermal collagen content and mitigates age-related stratum corneum thickening (Oizumi et al., 2024). Exercise-induced upregulation of sirtuins, particularly the NAD^+^-dependent deacetylase SIRT3, enhances mitochondrial biogenesis, energy metabolism, and oxidative stress resistance (Cheng et al., 2016; Zhou et al., 2022).

Resistance training also appears relevant. Human intervention studies report increased dermal thickness, improved elasticity, and enhanced upper-dermal extracellular matrix composition following structured programs (Nishikori et al., 2023).

Observational data further show that physically active older adults exhibit lower circulating CRP and IL-6 levels and improved metabolic health, correlating with delayed photoaging features (Young et al., 2022). Long-term exercisers maintain superior vascular and metabolic function, indirectly supporting cutaneous resilience (Curtis et al., 2015; Beavers et al., 2010). As exercise responses follow a dose–response relationship, excessive training without adequate recovery may induce catabolic and inflammatory states, highlighting the importance of balanced prescriptions.

Collectively, exercise exerts mechanistic effects on microcirculation, mitochondrial integrity, inflammatory tone, and dermal structure, contributing to a more resilient aging phenotype (Young et al., 2022).

### 3.3 Pillar III: stress regulation and neuro–immuno–skin aging

Psychological stress exerts measurable effects on skin biology through neuroendocrine, inflammatory, and oxidative pathways. Acute stress activates the hypothalamic–pituitary–adrenal (HPA) axis, increasing cortisol levels that correlate with impaired epidermal barrier recovery, reduced natural moisturizing factor content, and delayed repair after injury (Slavich, 2020). Activation of the HPA and sympathetic–adrenal–medullary axes elevates IL-6, CRP, and catecholamines, promoting inflammatory damage relevant to skin aging (Yegorov et al., 2020). Stress-induced mitochondrial dysfunction and impaired mitophagy generate persistent reactive oxygen species, accelerating cellular senescence (Ortiz et al., 2014; Passeron et al., 2021; Maarouf et al., 2019). Psychosocial stress has also been associated with hair aging phenotypes, including premature graying, within the broader exposome framework (Cedirian et al., 2025). Sustained sympathetic activation further impairs regenerative capacity, as murine models demonstrate depletion of melanocyte stem cells under chronic adrenergic signaling (Zhang et al., 2020).

Chronic stress amplifies these mechanisms. Prolonged glucocorticoid exposure disrupts mitochondrial function and increases oxidative damage in keratinocytes and fibroblasts (Slavich, 2020). Experimental data show reduced dermal collagen synthesis, increased matrix metalloproteinase activity (Yegorov et al., 2020), worsened transepidermal water loss, and delayed wound healing under sustained stress (Ortiz et al., 2014). Beyond direct cutaneous effects, chronic sympathetic arousal promotes maladaptive behaviors, including sleep disruption, poor diet, and physical inactivity, that synergistically accelerate systemic and skin aging (Baban and Morton, 2022).

Cohort studies report elevated circulating IL-6 and CRP levels in individuals with chronic psychological distress, consistent with systemic inflammaging (Hackett et al., 2012; Jaremka et al., 2013). In the skin, stress enhances mast cell activation, neurogenic inflammation, and exacerbation of inflammatory dermatoses (Peters, 2016). Stress-related alterations in microbial diversity and barrier function have also been described, aligning with broader exposome–microbiome interactions implicated in aging biology (Merra et al., 2024). Mitochondrial dysfunction and persistent inflammatory signaling observed in stress states parallel mechanisms of immune aging and extrinsic skin aging (Zheng et al., 2023). Conversely, mind–body interventions that attenuate sympathetic activation have been associated with reductions in cortisol and inflammatory markers, alongside improvements in immune regulation (Baban and Morton, 2022).

Together, these data indicate that chronic stress accelerates extrinsic skin aging by impairing barrier repair, sustaining oxidative and inflammatory signaling, and reducing regenerative stem cell reserves.

### 3.4 Pillar IV: sleep and circadian integrity in skin aging

Sleep is essential for epidermal regeneration, barrier repair, and immune regulation. Clinical evidence shows that insufficient or poor-quality sleep accelerates biological processes linked to skin aging. In a controlled human study, one night of total sleep deprivation prevented the normal decline in cerebrospinal fluid Aβ42 levels, suggesting impaired glymphatic clearance and increased oxidative and inflammatory burden (Ooms et al., 2014). Although derived from neurobiology, impaired clearance and oxidative stress are mechanistically relevant to fibroblast dysfunction and extracellular matrix degradation.

Chronic short sleep is associated with impaired barrier function. Individuals with poor sleep efficiency exhibit higher transepidermal water loss, reduced stratum corneum hydration, and delayed recovery after barrier disruption (Yoshizaki et al., 2017; Axelsson et al., 2010; Oyetakin-White et al., 2015). Beyond cutaneous effects, insufficient sleep correlates with cardiometabolic risk, immune dysregulation, chronic inflammation, and premature mortality, systemic alterations that amplify inflammatory and metabolic drivers of skin aging (Dedhia and Maurer, 2022).

Population-based studies further report elevated CRP and IL-6 levels in adults with habitual short sleep duration, consistent with systemic inflammaging and associated with accelerated wrinkle formation and reduced dermal collagen density (Besedovsky et al., 2012). Sleep deprivation also promotes maladaptive behaviors, including increased caloric intake and reduced physical activity, which further exacerbate inflammatory and metabolic stress.

Experimental models provide mechanistic support. Chronic sleep restriction increases oxidative stress, reduces antioxidant enzyme activity, and induces mitochondrial fragmentation in epidermal and dermal cells. Sleep fragmentation enhances glucocorticoid signaling, a pathway known to impair wound healing and collagen synthesis (Slavich, 2020). Conversely, sleep restoration reduces stress signaling and improves immune and metabolic regulation within weeks (Dedhia and Maurer, 2022).

Collectively, inadequate sleep accelerates skin aging by impairing barrier repair, amplifying oxidative and inflammatory pathways, and disrupting hormonal rhythms essential for nocturnal regeneration.

### 3.5 Pillar V: avoidance of deleterious exposures (sun, tobacco, pollution, alcohol)

Tobacco smoke, alcohol, environmental pollution, and solar radiation are major drivers of extrinsic skin aging through oxidative, inflammatory, and matrix-degrading pathways. Population data show that urban living and toxic occupational exposures are associated with higher skin aging grades (Nanzadsuren et al., 2022), while airborne toxicants activate oxidative and senescence pathways contributing to skin aging (Misra, 2020).

Solar radiation remains the predominant extrinsic factor. Photoaging is fundamentally driven by cumulative ultraviolet exposure, with its severity largely influenced by lifetime sun exposure, photoprotection behaviors, and environmental or occupational conditions. Although UVB induces direct DNA damage, UVA plays a central role in photoaging due to deeper dermal penetration and year-round exposure. UVA promotes oxidative stress, matrix metalloproteinase activation, collagen degradation, and pigmentary changes; infrared A and visible light further contribute to oxidative and pigmentary alterations (Gromkowska-Kępka et al., 2021). At the molecular level, ultraviolet radiation activates mitogen-activated protein kinase (MAPK) signaling cascades that stimulate activator protein-1 (AP-1) and nuclear factor kappa B (NF-κB), transcription factors that upregulate matrix metalloproteinases (MMPs), leading to collagen fragmentation and impaired dermal matrix repair. These mechanisms support the need for consistent, broad-spectrum photoprotection emphasizing UVA coverage.

Smoking independently accelerates skin aging. It reduces dermal collagen and elastin, impairs microcirculation, disrupts stem cell function, and increases facial wrinkling risk irrespective of sun exposure (Farage et al., 2008; Ernster et al., 1995). Mechanistically, tobacco smoke decreases perfusion, increases MMP-1 expression, induces keratinocyte DNA damage, and reduces antioxidant capacity (Loman et al., 2022). Both smoking and air pollution also stimulate melanogenesis, contributing to lentigines and dyspigmentation (Flament et al., 2018; Okada et al., 2013; Krutmann et al., 2017; Grether-Beck et al., 2021). Importantly, smoking cessation remains a highly actionable intervention, as physician counseling improves long-term quit rates (Brewer, 2022; Soeur et al., 2017; Ferrara et al., 2021).

Alcohol further disrupts cutaneous homeostasis. Ethanol metabolism generates acetaldehyde and reactive oxygen species, alters barrier lipids, and increases inflammatory cytokine release; clinically, high intake is associated with telangiectasias, reduced hydration, and exacerbation of inflammatory dermatoses (Liu and Chen, 2023).

Environmental pollutants such as particulate matter, polycyclic aromatic hydrocarbons, ozone, and ultraviolet radiation promote oxidative stress and aryl hydrocarbon receptor (AhR) activation, leading to barrier dysfunction and MMP-mediated collagen degradation (Parrado et al., 2019). PM2.5 exposure correlates with increased wrinkles and pigment spots, particularly when combined with UVA (“photo-pollution”) (Santamaria et al., 2025). Controlled human studies demonstrate rapid depletion of epidermal vitamin E and increased lipid peroxidation after ozone exposure (Thiele et al., 1998). Long-term traffic-related pollution exposure is associated with deeper wrinkles and pigmentary changes, independent of UV exposure (Brewer, 2022). At the molecular level, pollutants penetrate the stratum corneum, activate AhR signaling, and promote oxidative stress and matrix degradation in keratinocytes and fibroblasts (Papaccio et al., 2022).

Under real-life conditions, these stressors act synergistically. Combined ultraviolet and particulate exposure induces oxidative stress, mitochondrial dysfunction, and melanocyte senescence, reinforcing photo-pollution as a major driver of extrinsic aging (Martic et al., 2025).

### 3.6 Pillar VI: social connection, inflammation, and skin aging

Social relationships are a biologically relevant determinant of aging. Population data indicate that loneliness and insufficient social support are highly prevalent and clinically significant risk factors (Town et al., 2024). Reduced social participation and persistent loneliness are associated with increased all-cause mortality, cardiovascular risk and cognitive decline, features of accelerated biological aging (Krivanek et al., 2021). In a prospective study of older women, daily social contact was associated with nearly a 50% lower risk of dementia compared with limited social networks (Crooks et al., 2008). Social isolation is also linked to poorer treatment outcomes, longer hospitalizations, and increased cardiometabolic and psychiatric morbidity (Holt-Lunstad, 2022).

Psychobiological mechanisms provide explanatory support. Loneliness is associated with heightened stress reactivity and low-grade inflammation. Experimental studies show exaggerated IL-6 and cortisol responses to acute stress in socially isolated individuals (Hackett et al., 2012; Jaremka et al., 2013), while meta-analytic data link lower social integration with elevated inflammatory markers, including IL-6 and CRP (Uchino et al., 2012). Loneliness also predicts higher systolic blood pressure over time (Hawkley et al., 2010), and adverse social interactions contribute to cortisol dysregulation and inflammatory signaling (Ortiz et al., 2014). A large meta-analysis further demonstrated that stronger social connections are associated with approximately 50% greater survival likelihood (Holt-Lunstad et al., 2010).

Although direct dermatologic data remain limited, the systemic alterations associated with social isolation—chronic inflammation, cortisol dysregulation, vascular impairment, and immune aging—parallel pathways implicated in skin aging, including inflammaging and impaired tissue repair (Ortiz et al., 2014; Passeron et al., 2021; Maarouf et al., 2019). Within a Lifestyle Dermatology framework, strengthening social connection may therefore mitigate stress-related inflammatory load and support healthier skin aging trajectories. Collectively, these findings across lifestyle domains support a coherent model in which exposome-related and behavioral factors modulate systemic pathways that ultimately translate into skin-specific aging processes.

### 3.7 Clinical applications in dermatology

Lifestyle Dermatology principles can be incorporated into routine practice by assessing lifestyle-related exposures, counseling on modifiable behaviors, and integrating these strategies with established interventions, including retinoids, antioxidants, photoprotection, and procedural treatments. Objective phenotyping tools, such as high-resolution imaging, biomechanical assessment, transepidermal water loss measurement, and digital exposome tracking, may assist in monitoring treatment responses.

Integrating lifestyle-based strategies supports a preventive and patient-centered approach to skin aging. Longevity research indicates that lifestyle behaviors, circadian alignment, and targeted interventions can influence epigenetic aging and biological skin age (Haykal et al., 2025). Practical recommendations linking behavioral interventions with biological targets are summarized in Table 2.

**TABLE 2:** Lifestyle interventions, biological targets, and practical clinical recommendations in skin aging.

Intervention category	Behavioral intervention	Biological marker affected	Expected skin aging effect	Practical clinical recommendation
Nutritional modulation	Low-glycemic diet; ↑ polyphenols; Mediterranean-style patterns	↓ AGEs, ↓ NF-κB activation, ↓ oxidative stress	Improved collagen quality, reduced glycation	Recommend Mediterranean-style dietary patterns rich in polyphenols and fiber to reduce glycation burden and NF-κB–mediated inflammation; counsel reduction of high-glycemic foods known to accelerate collagen cross-linking
Exercise training	Moderate aerobic activity 3–5×/week; resistance training	↑ IL-15, ↑ mitochondrial biogenesis, ↓ CRP/IL-6	Improved dermal structure and resilience	Recommend structured aerobic and resistance programs to support mitochondrial biogenesis and dermal remodeling; monitor adherence in patients with sedentary lifestyles associated with accelerated aging phenotypes
Stress regulation	Mindfulness, breathing techniques, relaxation practices	↓ cortisol, ↓ IL-6/CRP, ↓ sympathetic tone	Reduced inflammaging, improved barrier repair	Integrate stress-reduction strategies to modulate cortisol-driven inflammatory signaling and support epidermal barrier repair, particularly in patients with stress-sensitive dermatoses
Sleep optimization	7–9 h of sleep; circadian alignment	↓ TEWL, ↓ cortisol, ↑ barrier recovery	Improved hydration, fewer wrinkles	Promote circadian-aligned sleep routines to enhance nocturnal barrier repair and reduce oxidative stress pathways linked to premature skin aging
Toxic avoidance	Smoking cessation; reduced alcohol and pollution exposure	↓ ROS, ↓ MMP-1, ↓ oxidative damage	Reduced photoaging and matrix degradation	Counsel smoking cessation and reduction of chronic pollutant exposure to limit ROS-driven MMP activation and collagen degradation
Photoprotection	Daily broad-spectrum photoprotection	↓ UVA-induced ROS, ↓ MMP activation	Slower photoaging, reduced pigmentary changes	Recommend daily broad-spectrum photoprotection with strong UVA coverage to prevent MAPK/AP-1–mediated MMP activation and cumulative dermal matrix degradation
Skincare practices	Gentle cleansing in morning; barrier-supportive routines; evening wash of exposed areas to remove pollutants	↑ Barrier lipids, ↓ TEWL, ↓ inflammation	Improved barrier integrity and repair	Encourage barrier-preserving cleansing routines and removal of environmental pollutants to reduce oxidative stress and support epidermal lipid homeostasis
Adjunctive dermocosmetics	Antioxidant, antipollution, mitochondrial-supporting formulations	↓ oxidative stress, ↑ mitochondrial function	Enhanced protection and repair	Consider formulations containing antioxidants and mitochondrial-supportive compounds to mitigate oxidative stress and enhance cellular repair pathways
Social connection enhancement	Supportive social interactions	↓ IL-6/CRP, ↓ stress reactivity	Improved repair capacity and resilience	Encourage supportive social engagement to reduce systemic inflammatory signaling associated with impaired repair and inflammaging

### 3.8 Future directions

Future research should prioritize controlled trials evaluating how structured exercise, targeted nutrition, and stress-modulation strategies influence objective biomarkers of skin aging, including collagen architecture, mitochondrial function, and inflammatory profiles. Early evidence that exercise enhances mitochondrial biogenesis (Oizumi et al., 2024) and that dietary patterns modulate oxidative and inflammatory pathways in dermal cells (Papaccio et al., 2022; Uribarri et al., 2010) supports the need for longitudinal studies in well-characterized aging cohorts. Senescence-targeting strategies and omics-based profiling may further enable personalized interventions (Hussein et al., 2025).

Multi-omics integration combining genomic, epigenomic, microbiome and exposome data offers a framework to clarify how behavioral exposures shape molecular aging trajectories, building on evidence linking epithelial barrier dysfunction, dysbiosis, and systemic inflammation (Pat et al., 2024). Coupling metabolomics with advanced skin imaging may help identify early, behavior-responsive biomarkers. In particular, epigenomic biomarkers such as DNA methylation clocks, metabolomic profiling of oxidative stress–related pathways, and microbiome-derived metabolite mapping represent promising translational tools for monitoring lifestyle-responsive mechanisms relevant to dermal matrix integrity and barrier function.

Digital exposome monitoring tools, including real-time tracking of UV exposure, air quality, sleep, and physical activity, may allow dynamic assessment of lifestyle–aging interactions (Haykal et al., 2026). Artificial intelligence–based biological age estimators derived from skin imaging and multi-omic datasets could support individualized aging risk prediction, extending findings that mitochondrial dysfunction, oxidative stress, and inflammaging are modifiable across the lifespan (Curtis et al., 2015). Emerging longevity research further highlights mitochondrial rejuvenation and senescence modulation as potential therapeutic targets (Berisha et al., 2025).

Together, these advances support a precision-oriented model in which mechanistic biomarkers and digital phenotyping guide personalized strategies to prevent or slow skin aging.

Despite increasing mechanistic and clinical evidence, several limitations should be considered. Much of the available data derives from experimental or observational studies that may not fully translate to human outcomes. Epidemiological findings may be influenced by confounding variables, including genetic predisposition and cumulative exposures, and many lifestyle measures rely on self-reported data, introducing potential reporting bias.

## 4 Conclusion

Skin aging is a biologically complex yet modifiable process shaped by cumulative environmental and behavioral exposures. The six pillars of Lifestyle Medicine, namely nutrition, physical activity, stress regulation, restorative sleep, avoidance of harmful exposures, and social connection, modulate shared mechanisms central to skin aging, including oxidative stress, chronic inflammation, mitochondrial dysfunction, and extracellular matrix remodeling.

Together, these findings support a mechanistically grounded framework in which lifestyle modification represents a clinically relevant strategy to prevent or slow the progression of skin aging.

## 5 Plain Language Summary

### 5.1 How daily habits may influence skin ageing

Skin ageing is a natural process that happens over time. However, it is not caused only by genetics. Our skin is also affected by lifelong exposure to sunlight, pollution, tobacco smoke, diet, stress and sleep patterns. The total impact of these environmental and lifestyle exposures is known as the “exposome”. Many of these factors are potentially modifiable.

In this review, we examined scientific evidence linking six key lifestyle areas: nutrition, physical activity, stress management, sleep, avoidance of harmful exposures (such as smoking and excessive sun exposure), and social connection, to biological processes involved in skin ageing. We focused on how these factors influence mechanisms such as inflammation, oxidative stress, mitochondrial function (how cells produce energy), and collagen breakdown.

We found that healthy lifestyle behaviours are consistently associated with biological pathways that may slow visible and structural features of skin ageing. For example, balanced diets may reduce oxidative damage, exercise may support skin blood flow and cellular energy function, good sleep may improve barrier repair, and stress reduction may decrease inflammation. Avoiding smoking and chronic sun exposure appears especially important.

Although most evidence is indirect and based on observational or mechanistic studies rather than large clinical trials specifically measuring skin ageing outcomes, the findings suggest that skin ageing is partly modifiable. Addressing lifestyle factors may complement dermatological treatments and support healthier skin across the lifespan.
